# Isolated Arterial Injury of Moynihan’s Hump During Laparoscopic Cholecystectomy: A Case Report With Video

**DOI:** 10.7759/cureus.104464

**Published:** 2026-02-28

**Authors:** Stanislav Litkevych, Artem Zelinskyi, Thaer Abdalla, Steffen Deichmann, Tobias Keck

**Affiliations:** 1 Surgery, University of Schleswig-Holstein, Luebeck, DEU

**Keywords:** bailout strategey cholecystectomy, • cvs: critical view of safety, isolated arterial injury cholecystectomy, laparoscopic cholecystectomy complication, moynihan’s hump (caterpillar hump)

## Abstract

Isolated arterial injury of major vessels during laparoscopic cholecystectomy is a rare complication, most commonly involving the right hepatic artery (RHA). Moynihan's hump (also known as "caterpillar hump") is a rare but significant anatomical variant that represents a major predisposing factor for iatrogenic vascular injuries during cholecystectomy. In this variant, the RHA follows a tortuous, U-shaped course within the hepatocystic triangle. This configuration often results in a short cystic artery, making the RHA itself highly vulnerable to accidental clipping or transection.

This case report demonstrates an isolated intraoperative injury to the RHA during laparoscopic cholecystectomy and its subsequent management. We discuss management strategies for arterial injury and preventive measures to avoid such complications.

Awareness of arterial variations in the hepatocystic triangle, along with routine implementation and proper understanding of the critical view of safety, could significantly reduce iatrogenic injuries during laparoscopic cholecystectomy. Knowing bailout strategies in laparoscopic cholecystectomy is crucial. The decision whether to reconstruct the RHA should be made on an individual, case-by-case basis.

## Introduction

The real incidence of an isolated arterial injury (IAI) during a laparoscopic cholecystectomy (LC) without a concomitant bile duct injury (BDI) has not been clearly and definitively quantified in large-scale studies. Pesce et al. reported that conversion to open surgery due to vascular lesions occurs in approximately 0%-1.9% of cases, with a mortality rate of about 0.02% [1]. According to Singla et al., the more devastating BDIs during this operation occur in 12%-61% of cases with concomitant vascular injuries [2].

One of the significant risk factors for arterial injury during LC is the presence of vascular variants of the cystic artery (CA) and right hepatic artery (RHA) [1]. Moynihan’s hump (MH), also called a caterpillar hump, is a U- or S-shaped configuration of the RHA in which the artery passes very close to the cystic duct and gallbladder in up to 12.9% of cases [1,3].

Ligation of the RHA could lead to ischemic necrosis, abscess, or atrophy of the right liver lobe and even a fulminant or chronic liver failure, especially in diseased livers, potentially necessitating liver transplantation. The partial injury can lead to a pseudoaneurysm, causing a delayed hemorrhage. Unexpected bleeding could compromise the surgeon's vision and could lead to concomitant injuries of the common bile duct and portal vein [4,5].

## Case presentation

**Figure 1:**
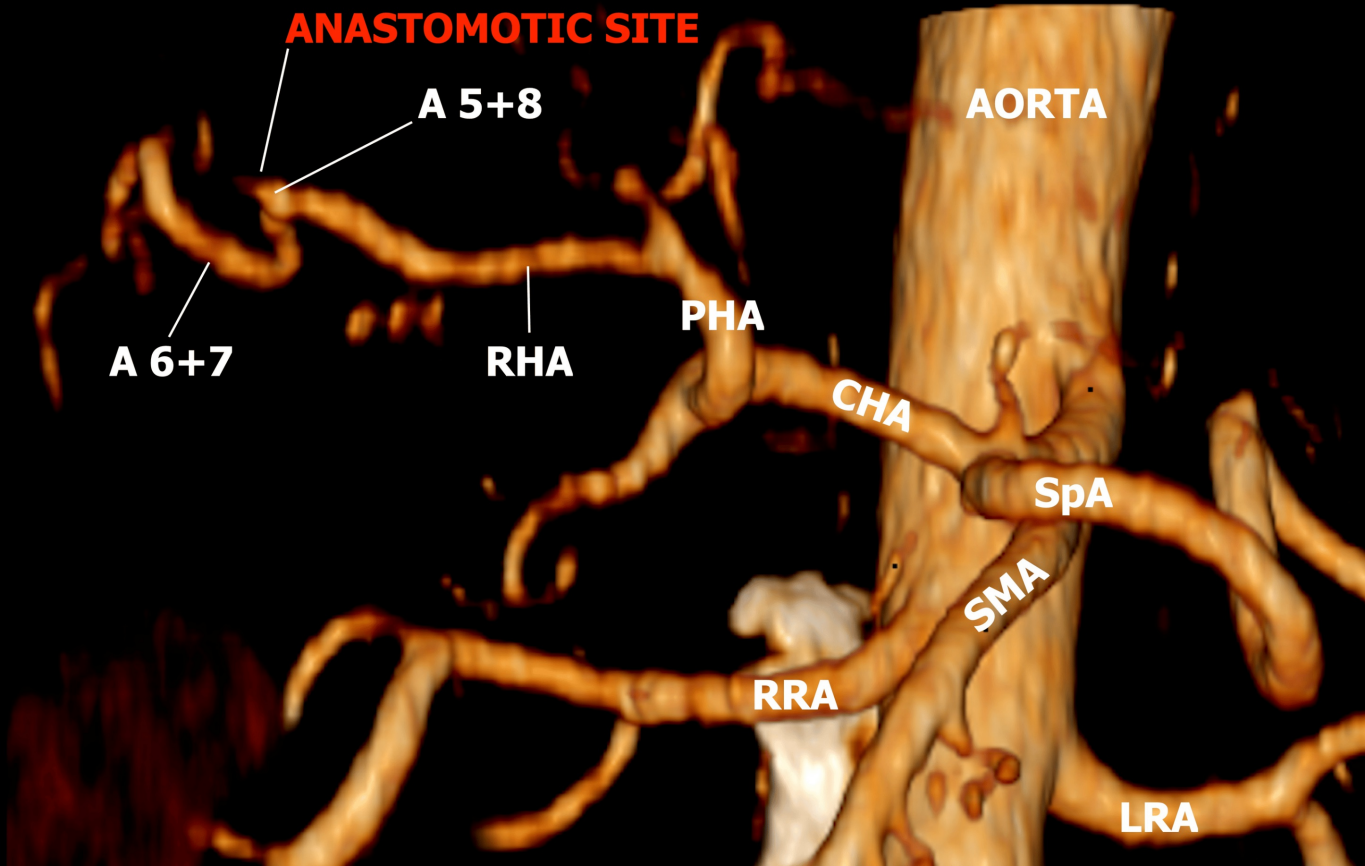
Absence of flow at the site of arterial reconstruction on CT angiography A 5+8: Anterior segmental artery of the liver; A 6+7: Posterior segmental artery; RHA: Right hepatic artery; PHA: Proper hepatic artery; CHA: Common hepatic artery; SpA: Splenic artery; RRA: Right renal artery; LRA: Left renal artery; SMA: Superior mesenteric artery.

Our report presents a case of LC complicated by an intraoperative isolated injury to an initially unrecognized "Moynihan's hump." The patient was a 50-year-old male with no significant comorbidities who underwent elective LC for symptomatic cholecystolithiasis. While approaching the "critical view of safety" (CVS), the MH was injured and clipped. After noticing ischemic changes in the liver segments V and VIII, the decision was made to reconstruct the damaged vessel after converting to a laparotomy. After the open reconstruction via end-to-end anastomosis using a magnification optic, there was a good pulsation of the artery intraoperatively. However, the immediate postoperative CT scan showed the absence of flow in the reconstructed vessel (Figure 1).

The occluded artery appeared to be a branch of the RHA, the anterior segmental artery, rather than the RHA itself. The postoperative course was uneventful, with no significant laboratory changes, and the patient was discharged on POD 3 (Video 1).

**Video 1:** Moynihan's/caterpillar hump injury during laparoscopic cholecystectomy

## Discussion

We discussed two key issues in this case: strategies to prevent vasculobiliary injuries (VBI) and the necessity of arterial reconstruction in the event of injury.

Regarding the first point, one of the most important measures to prevent misidentification injuries is the concept of CVS, as suggested by Strasberg et al. in 1995 [6]. Meanwhile, CVS is recommended as one of the most critical factors for overall safety during LC by SAGES (Society of American Gastrointestinal and Endoscopic Surgeons), as well as by international expert groups from Japan, Korea, Taiwan, the United States, India, and other societies and guidelines [7-9]. Despite this, most surgeons nowadays either fail to identify CVS descriptively or visually or claim to know CVS, without doing so in reality [8,10].

The CVS requires achieving three criteria [11]: the hepatocystic triangle should be cleared of fatty tissue, the cystic plate should be exposed in its lower third, and only two structures should enter the gallbladder (GB).

As CVS can be achieved in only 50% of cases [7], reasonable next steps after recognition of a difficult cholecystectomy include not proceeding with dissection without identification of anatomical landmarks (e.g., B-SAFE and R4U line), taking a time-out and calling for a second opinion, performing intraoperative cholangiography/sonography/indocyanine green (ICG) imaging, or employing bailout strategies such as subtotal cholecystectomy, top-down ("fundus first") approach, cholecystostomy, or converting to laparotomy [7,12].

**Figure 2:**
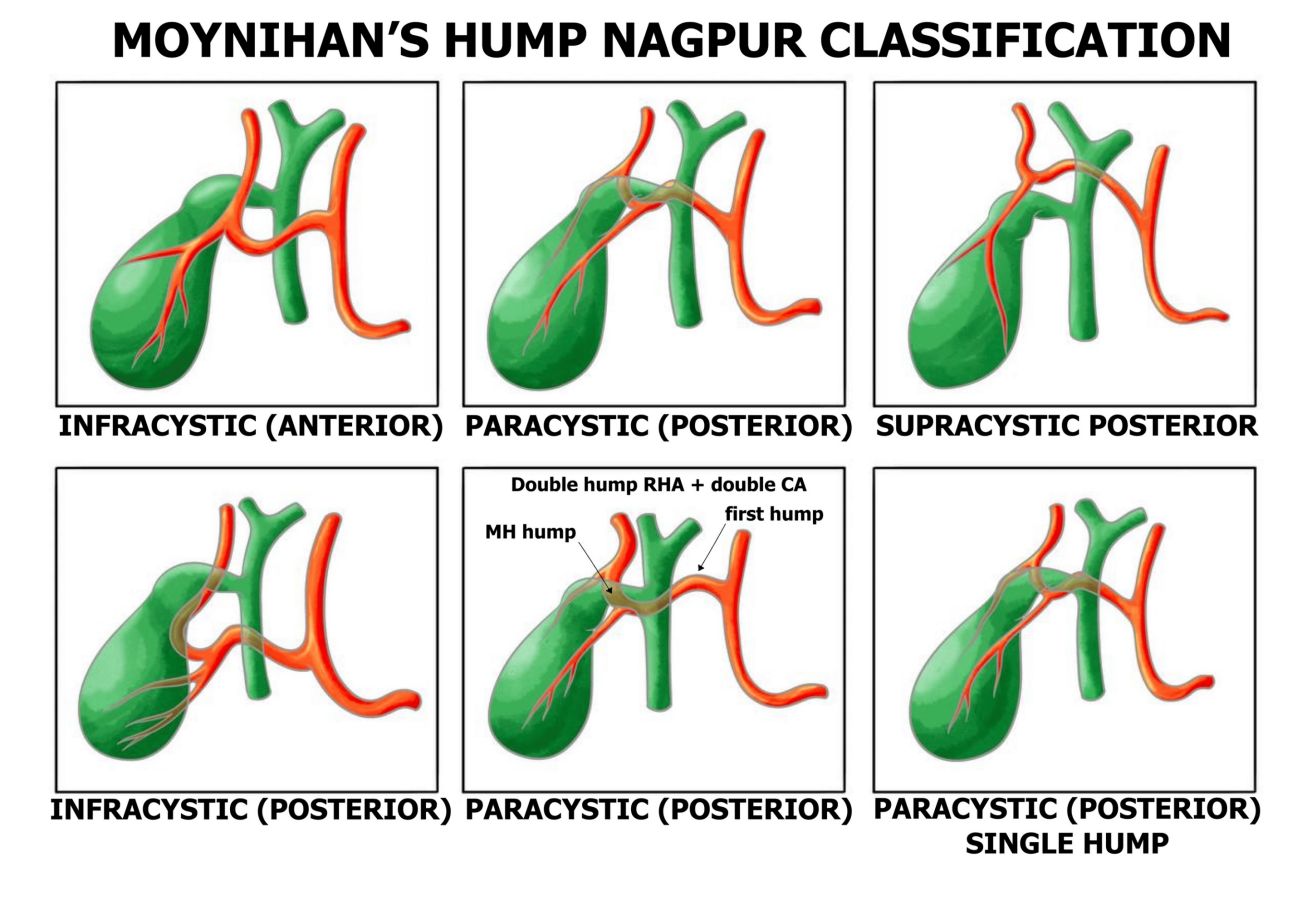
Moynihan’s hump - Nagpur classification Image credit: Sushych Hanna. Created with Procreate® (Savage Interactive Pty Ltd, Australia). Redrawn and modified based on the anatomical concepts described by [3]. Permission for reproduction/adaptation obtained from the original publisher, *Journal of Gastroenterology Research and Practice*. RHA: Right hepatic artery; CA: Cystic artery.

According to the Nagpur classification, variants of the MH in relation to the cystic duct include supra-, para-, and infracystic types, each of which can be anterior or posterior. The most common variants are the supracystic anterior type (52%), as observed in our case, and the paracystic posterior type (24%), as reported by Rahate ​​​​​​et al. [3] (Figure 2).

**Figure 3:**
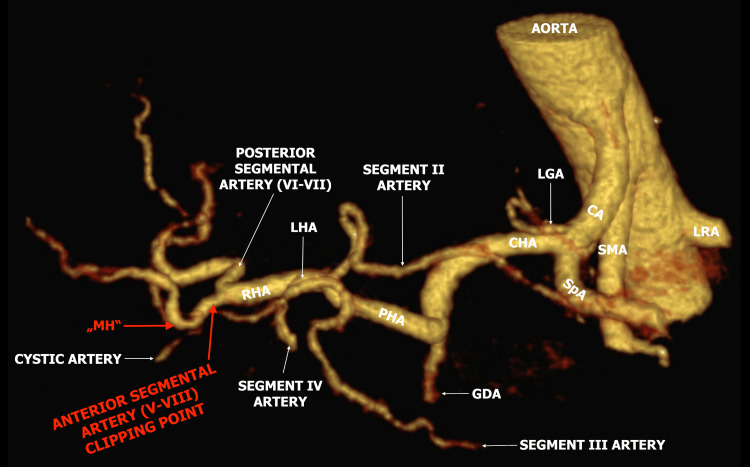
Older preoperative CT angiography scan MH: Moynihan’s hump; RHA: Right hepatic artery; LHA: Left hepatic artery; GDA: Gastroduodenal artery; CHA: Common hepatic artery; LGA: Left gastric artery; SpA: Splenic artery; CA: Celiac artery; SMA: Superior mesenteric artery; LRA: Left renal artery. (Courtesy of PD Jonas Ströder.)

Although the radiologic imaging for an LC is not a usual preoperative diagnostic, in our case, there was an older CT scan for other indications, where the MH was evident. It was obvious that we injured and clipped the anterior segment artery of segments V and VIII (Figure 3).

Regarding the second issue - whether to reconstruct the RHA after injury - the 2020 WSES guidelines state: "Systematic immediate repair of isolated injuries of the RHA is not recommended, and the benefit/risk ratio should be evaluated carefully. Weak recommendation, very low quality of evidence (GRADE 2C)" [7].

Only 10% of patients with RHA injury, even after combined VBI, develop clinically relevant hepatic ischemia. About 7% of patients had injury to the RHA or its branches after cholecystectomy, lacking signs of abnormalities of the liver or bile ducts based on cadaveric studies [13]. Burasakarn et al. [14] reported a small group of 10 patients in whom no reconstruction was performed after pancreatoduodenectomy for distal cholangiocarcinoma; no aforementioned complications were noted, provided the hilar bile duct was preserved. Similarly, in a small group of eight patients with tumor infiltration of the hepatic artery (HA) and gastroduodenal artery (GDA), the preoperative embolization of the HA to induce collateral blood flow and achieve a R0 resection without arterial reconstruction was performed, with uneventful outcomes [15]. Visual ischemic changes of the liver parenchyma after ligation of the RHA, as observed in our case, are not reliable predictors of the extent and severity of the future parenchymal damage, as they are subjective and do not take into account the following compensatory mechanisms [16].

The liver's vascular resilience relies on three main pillars: dual blood supply from arterial (25%-30%) and portal venous (70%-75%) sources, with each delivering 50% of oxygen; arterial collateral pathways and neovascularization through the hilar marginal artery (hilar shunt), hilar plexus (longitudinal shunt), interlobar collaterals, and phrenic, intercostal, and gastric arteries; and the "buffer response" of the portal venous system to hepatic arterial flow reduction [7,13,17].

RHA reconstruction should be considered in specific circumstances: partial injury (allowing less complex and more effective repair), transection proximal to the origin of the GDA, compromised liver (e.g., cirrhosis and portal vein thrombosis/hypertension), severe liver ischemia, simultaneous bile duct and/or portal vein injury, or when a surgeon with microvascular expertise (HPB (hepato-pancreato-biliary)/transplant/vascular/plastic) and high magnification optics is available, optimally at an HPB center [1,5,13,18].

Reconstruction techniques for HA include end-to-end or end-to-side anastomosis; arterial transposition due to vessel gap to the GDA, left gastric, or splenic arteries; graft interposition using autologous (saphenous, gonadal, inferior mesenteric veins, gastroepiploic, or radial artery) or prosthetic (e.g., polytetrafluoroethylene (PTFE) and Dacron) materials; and microsurgical techniques utilizing high magnification with microscope (6-15×) or high-power loops (6-8×) using monofilament non-absorbable suture (e.g., 8-0) [18,19].

Additionally, there is no single large series dealing exclusively with patency after iatrogenic HA reconstruction during LC. The available data mostly come from mixed case series (oncologic resections, liver transplantation, and complex HPB procedures) or smaller case reports. Tondolo et al. [20] evaluated a group of orthotopic liver transplantations in 532 patients. Among these patients, the incidence of HA thrombosis (HAT) was about 2.4%. Another strategy is to wait until the liver infarction is demarcated before performing the resection [13].

## Conclusions

Awareness of arterial variations, such as Moynihan's/caterpillar hump in the hepatocystic triangle, along with routine implementation and proper understanding of the CVS, could significantly reduce injuries during LC.

The decision to reconstruct the RHA should be made on an individual, case-by-case basis. In the majority of cases, the RHA can be ligated without significant consequences. If reconstruction is attempted, it should be performed by a surgeon experienced in microsurgical techniques, optimally using 6× or greater magnification, at an HPB center.
